# The Heterogenous Presentation of Hepatic Mucormycosis in Adults: A Case Report and Review of the Literature

**DOI:** 10.3390/jof11060408

**Published:** 2025-05-26

**Authors:** Hazim Allos, Rachel S. Hicklen, Takahiro Matsuo, Javier Adachi, Sebastian Wurster, Dimitrios P. Kontoyiannis

**Affiliations:** 1 Department of Infectious Diseases, Infection Control and Employee Health, The University of Texas MD Anderson Cancer Center, Houston, TX 77030, USA; hallos@mdanderson.org (H.A.); takahirom1226@gmail.com (T.M.); jaadachi@mdanderson.org (J.A.); stwurster@mdanderson.org (S.W.); 2 Research Medical Library, The University of Texas MD Anderson Cancer Center, Houston, TX 77030, USA; rshicklen@mdanderson.org

**Keywords:** mucormycosis, *Mucor* species, hepatic, liver, abscess

## Abstract

Hepatic mucormycosis is a rare but often fatal opportunistic fungal infection, primarily affecting immunocompromised patients. Herein, we report such a case from MD Anderson Cancer Center (Houston, TX, USA) and systematically review published cases in patients ≥ 19 years of age to better characterize clinical presentation, diagnostic challenges, and treatment outcomes of hepatic mucormycosis. Among the 40 identified cases (including ours), hematologic malignancies (55%) and solid organ transplantation (30%) were the most common underlying conditions. Fever (70%) and abdominal pain (63%) were the predominant symptoms. Imaging revealed multiple hepatic lesions in 72% of cases. Diagnosis was primarily based on histopathology (73%), whereas culture positivity was low (36%), underscoring the difficulty of pathogen isolation. Mucorales-active antifungal therapy was often delayed but eventually used in 85% of cases (all amphotericin B +/− Mucorales-active triazoles), while 45% underwent additional surgical intervention. Despite treatment, 1-year all-cause mortality remained high at 46%, with a trend towards lower mortality for those who underwent surgery compared to non-surgical management (35% vs. 55%, *p* = 0.334). These findings highlight the aggressive nature of hepatic mucormycosis and the importance of early recognition as well as the need for non-culture-based diagnostics and multimodal treatment approaches. Improved awareness and further research into optimized management strategies are crucial to improve the outcomes of this challenging infection.

## 1. Introduction

Mucormycosis (MCM), caused by Mucorales molds, is an increasingly encountered opportunistic infection which is often associated with poor outcomes, particularly in immunocompromised hosts [1]. *Rhizopus*, *Mucor*, and *Lichtheimia* species account for over 90% of MCM cases [2]. The pathogenesis of MCM is characterized by the fungi’s ability to invade blood vessels, leading to tissue necrosis and early hematogenous dissemination. This results in a wide array of clinical manifestations, including rhino-orbital, pulmonary, and cutaneous MCM [3]. Hepatic MCM, though uncommon [4] and not particularly well-studied in immunosuppressed patients, exemplifies the aggressive nature of these infections and the critical importance of early detection and comprehensive management strategies. Here, we present such a case along with a review of the literature on hepatic MCM.

## 2. Case Report

**Figure 1:**
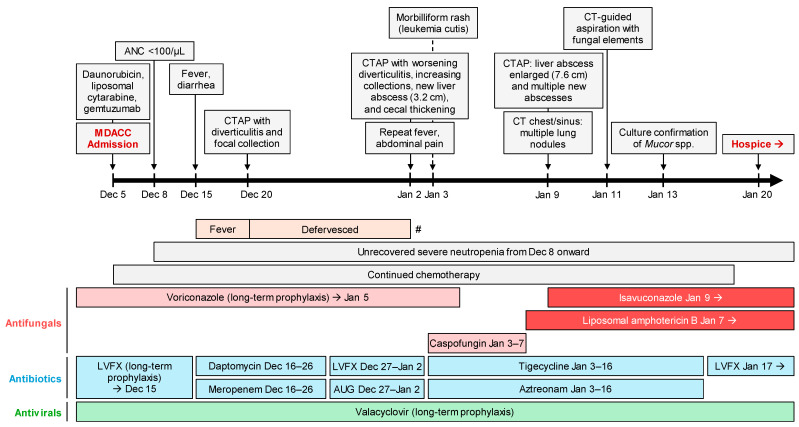
Timeline of clinical events and antimicrobial therapy. # indicates temporary recurrence of fever on 2 January 2024. Abbreviations: ANC, absolute neutrophil count; AUG, Augmentin; CT (AP), computed tomography (abdomen and pelvis); LVFX, Levofloxacin; MDACC, MD Anderson Cancer Center.

A 65-year-old male with relapsed acute myelomonocytic leukemia was treated with salvage azacitidine and venetoclax. Nonetheless, he continued to have active leukemia with hyperleukocytosis and 76% circulating blasts. For staging, the patient underwent a positron emission tomography scan on 4 December 2023, which showed widespread leukemic infiltration with multicompartment lymphadenopathy, widespread bone marrow fludeoxyglucose-18 (FDG) uptake, enlarged testicles with diffuse FDG uptake, and FDG uptake in the lungs and sigmoid colon. On the following day, the patient was admitted to MD Anderson Cancer Center (Houston, TX, USA) for further oncologic management. Bone barrow biopsy showed 95% blasts. He was then started on daunorubicin, liposomal cytarabine, and gemtuzumab for refractory disease (Figure 1).

Three days after initiating the new chemotherapeutic regimen, the patient developed profound neutropenia (<100 cells/µL) and began experiencing high-grade fevers and diarrhea on the 10th day of admission despite having been on long-term prophylaxis with levofloxacin, valacyclovir, and voriconazole (serum level: 1.7 μg/mL). Empiric intravenous antibiotic therapy with daptomycin and meropenem was initiated. A multiplex gastrointestinal pathogen panel, *Clostridium difficile* testing, serum *Aspergillus* galactomannan (Platelia™ *Aspergillus* antigen assay), and multiple blood cultures remained negative. Computed tomography of the abdomen and pelvis (CTAP) with contrast showed diverticular disease with a focus of diverticulitis and a sub-centimeter intramural fluid collection. The patient slowly defervesced and was recommended to finish a 10-day course of intravenous daptomycin and meropenem, and then transition to oral amoxicillin-clavulanate for 7 days, in addition to continued standard prophylaxis, including voriconazole.

**Figure 2:**
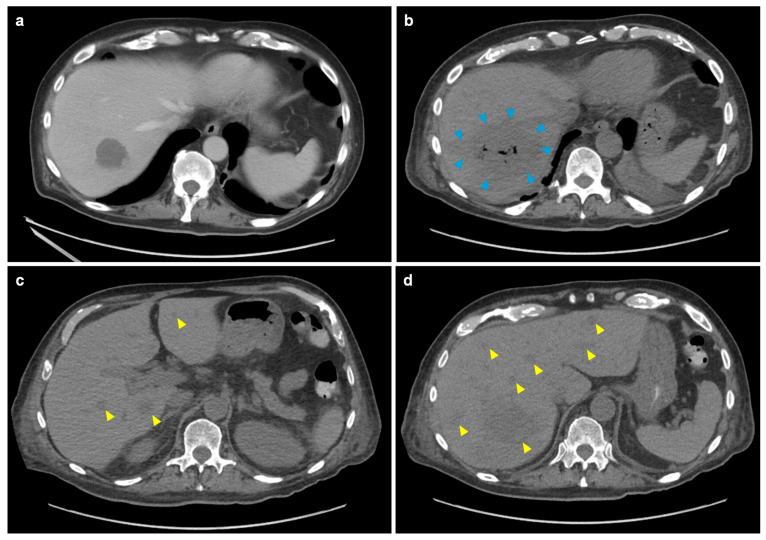
Evolution of hepatic lesions on abdominal computed tomography. (**a**) Image on 2 January 2024, displaying a solitary 3.2 cm abscess located in the right lobe of the liver. (**b**–**d**) Series of images from a follow-up scan on 9 January 2024 demonstrating significant progression of the initial abscess to 7.6 cm (blue arrowheads), with emergence of numerous new lesions scattered throughout the liver parenchyma (yellow arrowheads).

However, on 2 January 2024, the patient developed abdominal pain with recurrence of fever. The exam was significant for abdominal tenderness in the right upper quadrant and left lower quadrant. Repeat CTAP with contrast showed worsening diverticulitis and cecal thickening, increasing peri-sigmoid intramural collections, and a liver abscess measuring 3.2 cm in its largest dimension (Figure 2a). Additionally, liver function tests, previously normal, showed transient transaminitis with aspartate aminotransferase (AST) peaking near 1000 U/L, while alkaline phosphatase and bilirubin remained within the normal range. The following day, the patient developed a morbilliform rash on his trunk which eventually progressed to his extremities and face. The rash was confirmed to be leukemia cutis by punch biopsy.

Despite initiation of antibiotic therapy with tigecycline/aztreonam and antifungal therapy with caspofungin to cover possible hepatosplenic candidiasis, the patient’s symptoms persisted, although AST levels gradually resolved. However, follow-up CTAP on 9 January 2024 showed that the initial hepatic abscess had enlarged to 7.6 cm (Figure 2b). Additionally, multiple new abscesses were found (Figure 2c,d), along with new scattered pulmonary nodules measuring up to 5 mm. Suspecting disseminated MCM, liposomal amphotericin B (5 mg/kg, later increased to 7.5 mg/kg) and oral isavuconazonium sulfate (372 mg daily) were initiated.

Liver abscess culture and Calcofluor White staining were performed on hepatic material obtained via CT-guided aspiration. The culture grew *Mucor* species within ~48 h. A portion of the aspirate was submitted for histopathologic examination, which showed necrotic hepatic parenchyma containing invasive fungal elements consistent with Mucorales. Unfortunately, the patient’s pancytopenia precluded surgical intervention. In view of the patient’s refractory underlying disease, he eventually went home on hospice and died 21 days after the diagnosis of the liver abscess.

## 3. Literature Review

We performed a comprehensive search of the literature to identify published cases of MCM with liver involvement. Medline (Ovid), Embase (Ovid), Scopus, and Google Scholar were searched with no date restriction until 4 March 2025 using both natural language and controlled vocabulary terms for Mucorales, Mucormycosis, Zygomycosis, and liver/hepatic. After deduplication, 135 unique records were identified.

Only cases with confirmed hepatic MCM based on histopathology, microbiology, or molecular testing were included. Cases diagnosed solely on clinical suspicion, without such supporting evidence, were not included. Since pediatric cases may differ significantly in host factors and clinical course [5], we limited our review to adult patients (≥19 years) to ensure greater consistency in comorbidities, management strategies, and outcomes.

**Figure 3:**
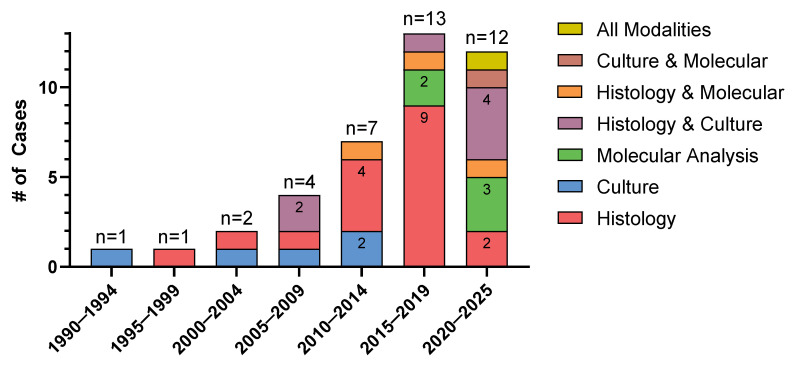
Number of published cases of hepatic mucormycosis from 1990 to 2025, stratified by diagnostic modality.

After applying these exclusions, we identified 39 eligible published cases plus our case reported in this manuscript, for a total of 40 cases included in our review (see Supplementary Table S1 for details) [6,7,8,9,10,11,12,13,14,15,16,17,18,19,20,21,22,23,24,25,26,27,28,29,30,31,32,33,34,35,36,37,38,39,40,41,42,43]. Overall, reported cases of hepatic MCM increased over time; 1990–1994 (n = 1), 1995–1999 (n = 1), 2000–2004 (n = 2), 2005–2009 (n = 4), 2010–2014 (n = 7), 2015–2019 (n = 13), and 2020–2025 (n = 12) (Figure 3).

The predominant underlying condition was hematological malignancy, observed in 22 of the 40 patients (55%), followed by solid organ transplantation (12/40 patients, 30%), including 1 patient who had both; 6 of the 22 patients with hematologic malignancy (29%) had a history of hematopoietic stem cell transplantation (Table 1). Among the 22 cases with hematologic malignancy, 12 (55%) reports specifically mentioned neutropenia and 4 (18%) either mentioned a diagnosis of necrotizing enterocolitis (NEC) or documented findings consistent with the syndrome based on symptomatology or imaging.

Eleven patients (28%) had documented antifungal prophylaxis, including six patients on fluconazole, two on voriconazole, two on posaconazole, and one on oral ketoconazole (Table 1). Seven (18%) patients were reported to not have received any antifungal prophylaxis, whereas antifungal prophylaxis was not mentioned in twenty-two (55%) cases.

Fever was the most common symptom associated with hepatic MCM, occurring in 28 out of the 40 cases (70%). Abdominal pain or tenderness was also frequently reported (25/40 patients, 63%), with 9 patients (23%) specifically experiencing right upper quadrant pain. Liver function test elevation was noted in 14 cases (35%) (Table 1).

Radiologic lesion characteristics were reported in 39 out of the 40 cases. Of those, 11/39 patients (28%) had solitary hepatic lesions, while 28/39 (72%) had multiple. The number of lesions ranged from 1 to 9, with a median of 1 and a mean of 2.0; however, these values are greatly skewed by incomplete reporting. Among patients with multiple lesions, the median number of lesions was 2.5 and the mean was 3.4. However, 20 of 28 cases did not specify the exact number of lesions. Among those with solitary lesions, size was reported in only five cases, ranging from 2.9 to 9.3 cm (median, 4.4 cm; mean, 5 cm) in the largest dimension.

Out of the 40 patients, 18 (45%) had suspected or proven extrahepatic manifestations, including additional intraabdominal lesions (Table 1). Among overlapping extrahepatic manifestations, the spleen was involved in seven (18%) patients and the bowel in eight (20%) patients, with one patient having involvement of both organs simultaneously. Notably, one patient with colonic pathology and splenic nodules underwent surgery, with histology confirming fungal invasion of the colon. Another patient had fungal invasion of the ileocecal valve and the same *Mucor* species later found in the liver abscess and also detected in stool surveillance prior to hepatic involvement. Another patient had biopsy-proven invasive disease in the esophagus and stomach, as well as positive peritoneal fluid cultures. Only 18 patients (45%) had imaging mentioned beyond the abdomen and pelvis, revealing lung lesions in 5 (13%), both lung and sinus lesions in 1 (3%), and brain lesions in 1 patient (3%). Nine patients (23%) showed no suspicious findings for extrahepatic involvement, while eleven (28%) had no additional intra-abdominal pathology noted and no extra-abdominal imaging performed. Two patients had no mention of either extra-abdominal imaging or pathology beyond the liver.

Overall, three cases (8%) of hepatic MCM were diagnosed postmortem on autopsy (histopathological examination). Of those, one patient had possible pulmonary involvement, one had splenic nodules, and another showed no extrahepatic involvement on autopsy.

Considering patients with overlapping modalities for confirmation of MCM, diagnosis was made by histopathology in 29 (73%) cases, molecular methods in 10 (25%), and culture in 14 (35%) (Table 1) (Figure 3). Notably, 25 of 39 cultures performed on the hepatic lesions (64%) showed no fungal growth. Organisms identified, including through molecular methods, included unspecified *Rhizopus* spp. (five), *Rhizopus microsporus* (three), *Mucor indicus* (three), *Rhizomucor pusillus* (two), *Rizhomucor miehei* (one), and unspecified *Rhizomcor*, *Lichtheimia*, and *Mucor* spp. (one each). Expectedly, the use of molecular diagnostics increased over time (2010–2014: n = 1; 2015–2019: n = 4; 2020–2025: n = 5), alongside traditional methods (Figure 3). Among the six cases where molecular methods were further specified, two were diagnosed by plasma metagenomic sequencing and four by tissue-based PCR methods.

Among 29 patients with available data, 8 (28%) patients had documented co-infection, including 4 patients with additional pathogens recovered from the sampled abscess (1 *Candida parapsilosis*, 1 *Pseudomonas aeruginosa*, 1 *Klebsiella* spp., and 1 polymicrobial combination of *Escherichia coli*, *Klebsiella pneumoniae*, *Enterococcus faecium*, and *Lactobacillus casei*). Additionally, three patients without bacterial recovery from the abscess had active bacteremia (one *Pseudomonas aeruginosa* and two *Klebsiella* spp.). One patient had bacterial peritonitis due to *Enterobacter aerogenes*.

In most cases, the initial working diagnosis was bacterial infection (n = 25, 63%), although four of these cases received additional antifungal coverage. Two cases (5%) were thought to be either fungal or bacterial. One case (3%) was thought to be either due to non-infectious etiologies or bacterial infection but received both antibiotics and antifungals. Four cases (10%) were initially thought to be due to non-infectious etiologies but one of them had received antibiotic therapy. Eight cases (20%) had no available data regarding initial differential diagnosis. Overall, the overwhelming majority of cases with available data (28/29, 97%) had received antibiotics as initial therapy, whereas only seven received antifungal therapy. Of these, only two patients initially received Mucorales-active antifungals.

Most patients (n = 34, 85%) eventually received Mucorales-active antifungal therapy after the diagnosis of hepatic MCM was made. All of them received amphotericin B-based regimens, often in combination with posaconazole (n = 7, 18%) or isavuconazonium sulfate (n = 2, 5%). Two patients did not receive Mucorales-active therapy and four had no available data. Eventually, 13 patients were transitioned to either of the two Mucorales-active triazoles as step-down monotherapy. The duration of antifungal therapy varied considerably, ranging from 60 days to 1 year, with two patients being prescribed lifelong suppressive therapy. Surgical intervention was undertaken in nearly half of the cases (n = 18, 45%).

**Figure 4:**
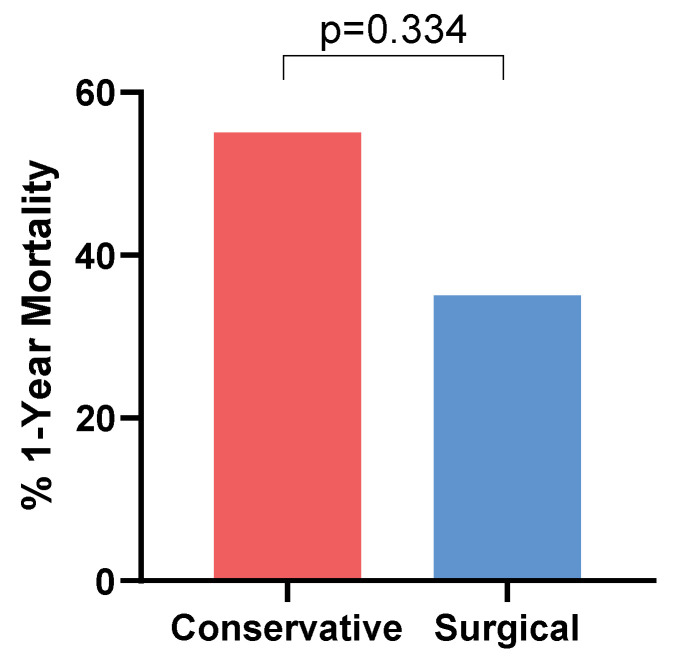
One-year mortality rates among patients treated with conservative versus surgical management. *p* value calculated using Fisher’s exact test.

Among the 39 cases with available data, 1-year all-cause mortality was 46% (18/39). Of these 18 patients, 17 died within 2 months of the diagnosis, with the remaining 1 passing 4 months post-diagnosis. Mortality rates tended to be lower in patients who underwent surgical therapy for hepatic MCM (6/17 with available data, 35%) than in those with non-surgical management (12/22, 55%), although this trend did not reach statistical significance (*p* = 0.334; Fisher’s exact test) (Figure 4).

On follow-up imaging, 11 patients (6 of whom had surgery) showed radiologic resolution of hepatic lesions, defined as complete disappearance of hepatic lesions on follow-up imaging. Notably, radiologic resolution of hepatic lesions did not occur in all patients deemed cured. Seven patients (two of whom had surgery) with persistent radiologic lesions showed clinical resolution, defined as clinical stability and absence of recurrence following completion of therapy. One patient who expired from an unrelated cause had clinical resolution, but imaging revealed persistent lesions. However, autopsy confirmed that the disease was inactive, at which point the patient was off therapy for several months. Among patients who were deemed clinically cured despite persistent lesions, two had a clearly documented reduction in lesion size with no comment on caliber or lesion number, while the remainder showed no radiographic change. No additional information was provided. In the 17 remaining patients who died within 2 months, radiographic resolution could not be evaluated. Four patients had no data available.

## 4. Discussion

Sino-pulmonary and sino-orbital disease are the most common clinical manifestations of MCM [44], whereas the gastrointestinal system is affected in fewer than 10% of MCM cases. Hepatic MCM is particularly unusual [45]. Of interest, in our review, 55% of patients had no evidence of disease outside of the liver. However, only 45% underwent extra-abdominal imaging, so the absence of extra-hepatic disease in some cases may reflect incomplete evaluation.

While our patient had a solitary liver lesion initially, the number of hepatic lesions may provide a diagnostic clue for MCM. In the reviewed cases, multiple lesions were noted on initial imaging for nearly three quarters of cases (72%). Similarly, the presence of multiple (>10) lesions has been reported as a distinguishing feature of pulmonary MCM compared to pulmonary aspergillosis, which typically presents with fewer lesions in high-risk patients with hematologic malignancies [46].

Our patient had breakthrough MCM in the setting of therapeutic serum voriconazole level and active hematologic malignancy, a classic scenario in this patient population [47]. Voriconazole intrinsically lacks activity against Mucorales due to reduced binding affinity to the structurally distinct 14α-demethylase enzyme [48]. Its use may suppress susceptible fungi, allowing resistant organisms such as Mucorales to proliferate. Indeed, up to 32% of all breakthrough fungal infections while on voriconazole have been attributed to Mucorales [47,49]. Experimental studies have shown increased virulence of Mucorales following preexposure to voriconazole [50]. Breakthrough infections on Mucorales-active triazoles have also been reported, but at significantly lower rates.

In our case, it further seemed that the inciting event for hepatic manifestations may have been NEC, specifically diverticulitis. NEC manifests as a severe inflammatory and necrotizing condition of the lower intestinal tract, predominantly in patients with hematologic malignancies undergoing aggressive chemotherapy [51]. Pathogens implicated in NEC include a polymicrobial spectrum, most commonly *Pseudomonas aeruginosa*, *Escherichia coli*, *Klebsiella* spp., *viridans Streptococci*, *Enterococci*, and anaerobes such as *Bacteroides*; however, fungi such as *Candida* are implicated as well [52]. Far less commonly, on autopsy, molds such as *Aspergillus* and Mucorales are found but are either clinically silent or are otherwise rarely considered for empiric treatment [53]. While specific risk factors for fungal NEC remain unclear, contaminated homeopathic medicines and supplements, certain foods including dried bread and fermented milk products, and pica syndrome have been identified as risk factors for gastrointestinal MCM [28,54]. No specific risk factor was identified in our patient, despite careful review of his clinical history.

Colonization of the gut by fungi, including Mucorales, can lead to dissemination either via the bloodstream or directly to the liver and spleen via the portal vein [55]. In our review, several cases seemed to have possibly originated from an initial gastrointestinal source, half of which were leukemia patients with descriptions consistent with NEC. Two of these patients had histologic confirmation of *Mucor* invasion of the bowel. Simultaneous involvement of the liver and spleen was observed in 18% of the reported cases, resembling the presentation of hepatosplenic candidiasis [56]. Additionally, three patients had abscesses co-infected with Gram-negative bacteria and one with *Candida*, while another three developed Gram-negative bacteremia, and one presented with Gram-negative peritonitis—further supporting a gastrointestinal source.

This review documents an apparent rise in hepatic MCM diagnoses over the past three decades, likely reflecting not only increased awareness, a growing immunocompromised population, and improved diagnostic modalities, but also potential publication bias, selective reporting, and methodological heterogeneity across eras. Despite recent diagnostic advances [57], diagnosing Mucorales remains challenging, as these organisms are notoriously difficult to culture, with negative culture results reported in up to 75% of cases [3].

In this review, molecular diagnostics seem to have been utilized more frequently in the last decade (Figure 3). Notably, 25% of cases had diagnosis of hepatic MCM via molecular methods, including 13% where molecular testing was the sole confirmatory modality. In recent years, molecular diagnostics have emerged as valuable tools to address this limitation. Mucorales-specific PCR assays, in particular, have shown high sensitivity—especially in serum and bronchoalveolar lavage samples—and may facilitate earlier detection compared to traditional culture or histopathology [58]. While the impact of Mucorales PCR on mortality outcomes remains under investigation, emerging evidence suggests that it may improve survival when used as a preemptive screening tool in high-risk populations [59,60]. Furthermore, PCR dynamics may have prognostic significance, as persistent PCR positivity after antifungal initiation correlates with poorer survival [60,61], while lower baseline fungal DNA burden has been linked to improved short-term survival [62]. Additionally, mold plasma cell-free DNA (cfDNA) PCR has been reported to be highly concordant with invasive specimen fungal test results in invasive mold infections, including MCM [63]. If validated by further studies, noninvasive tools such as this may reduce the need for invasive procedures in immunocompromised patients with suspected MCM, particularly given that invasive sampling and testing may not always be feasible in this at-risk population.

Notably, in two cases, metagenomic sequencing from peripheral blood enabled diagnosis, with one of these cases being histologically confirmed. Next-generation sequencing methods using cfDNA, such as metagenomic and whole-genome sequencing, offer broad fungal identification and hold potential for resistance profiling. While challenges related to cost, turnaround time, and database completeness remain, continued advancements are making these techniques increasingly viable for clinical use [64,65]. Therefore, these methods may be a valuable consideration when clinical suspicion is high.

Antifungal therapy of MCM typically consists of amphotericin B-based therapy, often combined with a Mucorales-active triazole such as posaconazole or isavuconazonium sulfate, though evidence for combination therapy is limited. Step-down therapy consists of monotherapy with these triazoles [3]. Duration varies and is highly individualized as spread of infection, activity of underlying diseases, management of comorbidities, timing of initiation of effective antifungal therapy, and feasibility of surgical intervention vary greatly [66]. Surgical debridement is considered a fundamental component of treatment alongside effective antifungal therapy and has been associated with improved outcomes [67]. This review supports this notion, demonstrating a trend toward a lower mortality rate among patients who underwent surgical intervention (35% vs. 55%), although this might reflect selection biases. Importantly, as noted above, some patients who were considered clinically cured did not achieve complete resolution of their lesions, a conundrum that is also observed in other anatomical sites of infection and, therefore, should not be the sole factor guiding treatment decisions.

## 5. Conclusions

In this manuscript, we presented a rare case of hepatic MCM in a neutropenic patient with active leukemia, along with a comprehensive review of published hepatic MCM cases. Our review illustrates the heterogeneous clinical manifestations of this rare entity and the formidable challenges posed by these infections, including a plethora of extra-hepatic manifestations, frequent initial misdiagnosis, poor recovery of Mucorales on culture, and high mortality. Earlier non-culture-based diagnostic modalities, such as PCR, might improve the outcome. However, while the underlying disease commonly remains the main driver of prognosis, surgical intervention seems to provide a beneficial impact.

## Figures and Tables

**Table 1:** Summary of the 40 reported hepatic MCM cases. Unless stated otherwise, numbers and percentages are provided.

Demographics	
Sex, male	29 (73%)
Age, median years (range)	39 (19–75)
Underlying conditions ^a^	
Hematologic malignancy	22 (55%)
Acute myeloid leukemia	10
Acute lymphoblastic leukemia	5
Lymphoma	3
Myelodysplastic syndrome	2
Chronic lymphocytic leukemia	1
Myelofibrosis	1
Solid organ transplant	12 (30%)
Renal	5
Liver	5
Pulmonary	1
Pancreatic and renal	1
Presenting symptoms	
Fever	28 (70%)
Abdominal pain	25 (63%)
Elevated liver function test	14 (35%)
Antifungal prophylaxis	11 (28%)
Fluconazole	6
Voriconazole	2
Posaconazole	2
Ketoconazole	1
Hepatic imaging ^b^	
Solitary lesion	11/39 (28%)
Multiple lesions	28/39 (72%)
Disseminated disease	
Clinical and/or radiologic suspicion of extrahepatic manifestations ^c^	18 (45%)
No suspicious findings for extrahepatic involvement	9 (23%)
No extra-hepatic intra-abdominal pathology noted and no extra-abdominal imaging performed	11 (28%)
No data on extra-hepatic manifestations	2 (5%)
Diagnosis of mucormycosis (some patients had multiple overlapping methods)	
Histopathology	29 (73%)
Molecular	10 (25%)
Culture	14 (36%)
Mucorales genus identified (including via molecular methods)	
*Rhizopus*	8 (20%)
*Rhizomucor*	4 (10%)
*Mucor*	4 (10%)
*Lichtheimia*	1 (3%)
Co-infections	
Co-pathogen identified ^d^	8/29 (28%)
Therapy	
Mucorales-active antifungals as part of initial therapy	2 (5%)
Mucorales-active antifungal therapy at some point during hospitalization	34 (85%)
Surgery	18 (45%)
Outcomes	
Radiographic resolution ^e^	11/19 (58%)
1-year all-cause mortality ^f^	18/39 (46%)

^a^ One patient had both hematologic malignancy and solid organ transplant. ^b^ One patient had no data available. ^c^ Includes 14 patients with extrahepatic lesions documented on imaging: 5 spleen, 2 lung and bowel, 2 lung, 1 brain, 1 lung and sinus, 1 lung and spleen, 1 bowel and spleen, 1 esophagus, stomach and peritoneum. ^d^ Data available from 29 patients. ^e^ Data was reported from 36 patients; however, radiographic resolution could not be assessed in 17 additional patients due to short-term mortality within 2 months. ^f^ One patient had no data available.

## Data Availability

All relevant data is contained in the manuscript and Supplementary Materials.

## Supplementary Materials

The following supporting information can be downloaded at https://www.mdpi.com/article/10.3390/jof11060408/s1: Table S1: Summary of clinical characteristics of the 40 hepatic MCM cases.

## Abbreviations

The following abbreviations are used in this manuscript:

AST	aspartate aminotransferase
cfDNA	Cell-Free Deoxyribonucleic Acid
CTAP	computed tomography of the abdomen and pelvis
FDG	fludeoxyglucose-18
MCM	mucormycosis
NEC	necrotizing enterocolitis
PCR	polymerase chain reaction
spp.	species

## References

[1] Spellberg B., Kontoyiannis D.P., Fredricks D., Morris M.I., Perfect J.R., Chin-Hong P.V., Ibrahim A.S., Brass E.P. (2012). Risk Factors for Mortality in Patients with Mucormycosis. Med. Mycol..

[2] Uppuluri P., Alqarihi A., Ibrahim A.S. (2021). Mucormycoses. Encyclopedia of Mycology.

[3] Alqarihi A., Kontoyiannis D.P., Ibrahim A.S. (2023). Mucormycosis in 2023: An Update on Pathogenesis and Management. Front. Cell Infect. Microbiol..

[4] Henry B., Lefevre Utile A., Jaureguiberry S., Angoulvant A. (2025). Gastrointestinal and Intra-Abdominal Mucormycosis in Non-Haematological Patients—A Comprehensive Review. J. Fungi.

[5] Francis J.R., Villanueva P., Bryant P., Blyth C.C. (2018). Mucormycosis in Children: Review and Recommendations for Management. J. Pediatr. Infect. Dis. Soc..

[6] Abboud C.S., Bergamasco M.D., Baía C.E.S., Lallée M.P., Zan A.S.C., Zamorano M.M., Pereira O.I., Mies S. (2012). Case Report of Hepatic Mucormycosis After Liver Transplantation: Successful Treatment with Liposomal Amphotericin B Followed by Posaconazole Sequential Therapy. Transplant. Proc..

[7] Aceves-Sánchez B., Rojas-Castañeda E., Ponce-de-León A., López-Iñiguez Á., Rangel-Cordero A., Sánchez E., Salgado-Nesme N., González-Lara M.F. (2024). Mucormycosis after Liver Transplant: Case Series and Literature Review. Med. Mycol. Case Rep..

[8] Bernardo R.M., Gurung A., Jain D., Malinis M.F. (2016). Therapeutic Challenges of Hepatic Mucormycosis in Hematologic Malignancy: A Case Report and Review of the Literature. Am. J. Case Rep..

[9] Busca A., Marmont F., Locatelli F., Limerutti G., Sorrentino M.T., Barbui A., Patrono D., Salizzoni M., David E., De Rosa F. (2010). Combined Antifungal Therapy, Iron Chelation and Surgical Resection as Treatment of Hepatic Zygomycosis in a Patient with Haematological Malignancy. Mycoses.

[10] Chaudhary R.J., Choudhary N.S., Saraf N., Gautam D., Piplani T., Thiagrajan S., Bhangui P., Saigal S., Rastogi A., Soin A.S. (2020). Delayed Graft Dysfunction Due to Invasive Hepatic Mucormycosis After Living Donor Liver Transplantation. J. Clin. Exp. Hepatol..

[11] Czapka M., Santos C.A.Q., Proia L.A. (2019). Isolated Hepatic Mucormycosis in the Early Post-Transplant Period: A Case Report and Literature Review. OBM Transplant..

[12] Deb S., Savio J., Padaki P.A. (2022). P181 Mucor in the Land of the Liver: A Case Report. Med. Mycol..

[13] Ganesh K., Abraham M.A., Kumar J.S., Simon S. (2021). Invasive Fungal Diseases in Renal Transplantation—Case Series. Indian. J. Transplant..

[14] Le Gac G., Allyn J., Coolen-Allou N., Lagrange-Xelot M., Fernandez C., Allou N., Hoarau G. (2017). Mucormycose Hépatique à Rhizopus Microsporus: Description d’un Cas. Med. Mal. Infect..

[15] Gali S., Rukmangadha N., Prayaga A., Kumar V.S. (2019). Hepatic Mucormycosis in a Renal Transplant Recipient: A Rare Presentation. Indian. J. Transplant..

[16] Gillrie M., Chow B., Griener T., Johnson A., Church D. (2023). Hepatosplenic Mucormycosis Due to Rhizomucor Pusillus Identified by Panfungal PCR/Sequencing of Ribosomal ITS2 and LSU Regions in a Patient with Acute Myelogenous Leukemia: A Case Report. J. Assoc. Med. Microbiol. Infect. Dis. Can..

[17] Grabau M., Pandya S., Nanjappa S., Shenoy R., Aslam S., Greene J.N. (2017). Liver Abscess in Patients with Leukemia and Prolonged Neutropenia. Infect. Dis. Clin. Pract..

[18] Karigane D., Kikuchi T., Sakurai M., Kato J., Yamane Y., Hashida R., Abe R., Hatano M., Hasegawa N., Wakayama M. (2019). Invasive Hepatic Mucormycosis: A Case Report and Review of the Literature. J. Infect. Chemother..

[19] Khan A.A., Kumaran V., Jain D., Siraj F., Aggarwal S. (2013). Hepatic Mucormycosis in a Patient of Acute Lymphoblastic Leukemia: A Case Report with Literature Review. Indian. J. Hematol. Blood Transfus..

[20] Lalwani S., Govindasamy M., Gupta M., Siraj F., Varma V., Mehta N., Kumaran V., Mohan N., Chopra P., Arora A. (2012). Gastrointestinal Mucormycosis—Four Cases with Different Risk Factors, Involving Different Anatomical Sites. Indian. J. Gastroenterol..

[21] Li K.-W. (2010). Hepatic Mucormycosis Mimicking Hilar Cholangiocarcinoma: A Case Report and Literature Review. World J. Gastroenterol..

[22] Mehta C., Ali M.T., Mehta Y., Anand J.S., George J.V. (2016). A Rare Case of Isolated Hepatic Mucormycosis in Association with Hemophagocytosis Syndrome. J. Acute Med..

[23] Mekeel K.L., Hemming A.W., Reed A.I., Matsumoto T., Fujita S., Schain D.C., Nelson D.R., Dixon L.R., Fujikawa T. (2005). Hepatic Mucormycosis in a Renal Transplant Recipient. Transplantation.

[24] Mezhir J.J., Mullane K.M., Zarling J., Satoskar R., Pai R.K., Roggin K.K. (2009). Successful Nonoperative Management of Gastrointestinal Mucormycosis: Novel Therapy for Invasive Disease. Surg. Infect..

[25] Padmanabhan S., Battiwalla M., Hahn T., Ball D., Paplham P., Brown K., Segal B.H., McCarthy P., Almyroudis N.G. (2007). Two Cases of Hepatic Zygomycosis in Allogeneic Stem Cell Transplant Recipients and Review of Literature. Transplant. Infect. Dis..

[26] Parmentier C., Cruz-Martinez R., Quintero-Quintero M., Vilatobá M. (2024). Mucormycosis in Liver Allograft Following Transplant for Secondary Biliary Cirrhosis. Exp. Clin. Transplant..

[27] Peng X., Wei Z., Wang L., Cheng J. (2023). Invasive Splenic Mucormycosis Due to Rhizopus Microsporus during Chemotherapy for Acute Monocytic Leukemia: A Case Report and Literature Review. Front. Oncol..

[28] Oliver M.R., Van Voorhis W.C., Boeckh M., Mattson D., Bowden R.A. (1996). Hepatic Mucormycosis in a Bone Marrow Transplant Recipient Who Ingested Naturopathic Medicine. Clin. Infect. Dis..

[29] Randi B.A., de Oliveira V.F., Rapozo M.M., Higashino H.R., Barbaro del Negro G.M., Chaves Magri M.M., Rocha V., Costa S.F. (2025). Fatal Hepatic Mucormycosis in an Allogeneic Hematopoietic-Stem Cell Transplanted Patient: Case Report of a Rare Presentation and Review of the Literature. J. Infect. Chemother..

[30] Reis F.P.d., Campos S.V., Aiello V.D., Duarte M.I.S., Samano M.N., Pego-Fernandes P.M. (2019). Gastrointestinal Mucormycosis Post Lung Transplantation. Braz. J. Infect. Dis..

[31] Rivero A., Shaughnessy M., Oswald J., Goodhope N., Oethinger M. (2025). Gastrointestinal Mucormycosis by Mucor Indicus: A Report of Two Cases. Med. Mycol. Case Rep..

[32] Sahu K.K., Yanamandra U., Kakkar N., Malhotra P. (2019). Rare Presentation of Mucormycosis in Aplastic Anaemia: Isolated Hepatic Mucormycosis. Mycopathologia.

[33] Schneider M., Kobayashi K., Uldry E., Demartines N., Golshayan D., Halkic N. (2021). Rhizomucor Hepatosplenic Abscesses in a Patient with Renal and Pancreatic Transplantation. Ann. R. Coll. Surg. Engl..

[34] Shah M., Nel J., Almansouri A., Van Duin D., Gerber D.A. (2019). Combined Medical and Surgical Management of Hepatic Mucormycosis in an Adult with Acute Myeloid Leukemia: Case Report and Review of the Literature. Mycopathologia.

[35] Shen M., Li Q., Zeng Z., Han D., Luo X. (2023). Mucor Indicus Caused Disseminated Infection Diagnosed by Metagenomic Next-Generation Sequencing in an Acute Myeloid Leukemia Patient: A Case Report. Front. Cell Infect. Microbiol..

[36] Su H., Thompson G.R., Cohen S.H. (2012). Hepatic Mucormycosis with Abscess Formation. Diagn. Microbiol. Infect. Dis..

[37] Suh I.W., Park C.S., Lee M.S., Lee J.H., Chang M.S., Woo J.H., Lee I.C., Ryu J.S. (2000). Hepatic and Small Bowel Mucormycosis after Chemotherapy in a Patient with Acute Lymphocytic Leukemia. J. Korean Med. Sci..

[38] Borg F.T., Kuijper E.J., Van Der Lelie H. (1990). Fatal Mucormycosis Presenting as an Appendiceal Mass with Metastatic Spread to the Liver during Chemotherapy-Induced Granulocytopenia. Scand. J. Infect. Dis..

[39] Van Sickels N., Hoffman J., Stuke L., Kempe K. (2011). Survival of a Patient with Trauma-Induced Mucormycosis Using an Aggressive Surgical and Medical Approach. J. Trauma Inj. Infect. Crit. Care.

[40] Trenker C., Dohse M., Metzelder S., Rexin P., Mariss J., Goerg C. (2016). 71-Year-Old Patient with Chronic Lymphocytic Leukemia (CLL) and Hypoechoic Nodular Spleen and Liver Lesions with Fatal Outcome: Presentation of Mucormycosis in B-Mode Imaging and Contrast-Enhanced Ultrasound (CEUS). Ultrasound Int. Open.

[41] George Tsaousis A.K. (2000). Liver and Brain Mucormycosis in a Diabetic Patient Type II Successfully Treated with Liposomial Amphotericin, B. Scand. J. Infect. Dis..

[42] Vikum D., Nordøy I., Torp Andersen C., Fevang B., Line P.D., Kolrud F.K., Aukrust P., Buanes T. (2017). A Young, Immunocompetent Woman with Small Bowel and Hepatic Mucormycosis Successfully Treated with Aggressive Surgical Debridements and Antifungal Therapy. Case Rep. Infect. Dis..

[43] Yasmeen S., Waqas O., Munir J., Sultan F., Hameed A. (2017). Hatosplenic Mucormycosis Post Autologous Stem Cell Transplant. Pak. J. Med. Sci..

[44] Lanternier F., Dannaoui E., Morizot G., Elie C., Garcia-Hermoso D., Huerre M., Bitar D., Dromer F., Lortholary O. (2012). A Global Analysis of Mucormycosis in France: The RetroZygo Study (2005–2007). Clin. Infect. Dis..

[45] Roden M.M., Zaoutis T.E., Buchanan W.L., Knudsen T.A., Sarkisova T.A., Schaufele R.L., Sein M., Sein T., Chiou C.C., Chu J.H. (2005). Epidemiology and Outcome of Zygomycosis: A Review of 929 Reported Cases. Clin. Infect. Dis..

[46] Chamilos G., Marom E.M., Lewis R.E., Lionakis M.S., Kontoyiannis D.P. (2005). Predictors of Pulmonary Zygomycosis versus Invasive Pulmonary Aspergillosis in Patients with Cancer. Clin. Infect. Dis..

[47] Boutin C.-A., Durocher F., Beauchemin S., Ziegler D., Abou Chakra C.N., Dufresne S.F. (2024). Breakthrough Invasive Fungal Infections in Patients with High-Risk Hematological Disorders Receiving Voriconazole and Posaconazole Prophylaxis: A Systematic Review. Clin. Infect. Dis..

[48] Caramalho R., Tyndall J.D.A., Monk B.C., Larentis T., Lass-Flörl C., Lackner M. (2017). Intrinsic Short-Tailed Azole Resistance in Mucormycetes Is Due to an Evolutionary Conserved Aminoacid Substitution of the Lanosterol 14α-Demethylase. Sci. Rep..

[49] Lionakis M.S., Lewis R.E., Kontoyiannis D.P. (2018). Breakthrough Invasive Mold Infections in the Hematology Patient: Current Concepts and Future Directions. Clin. Infect. Dis..

[50] Lamaris G.A., Ben-Ami R., Lewis R.E., Chamilos G., Samonis G., Kontoyiannis D.P. (2009). Increased Virulence of Zygomycetes Organisms Following Exposure to Voriconazole: A Study Involving Fly and Murine Models of Zygomycosis. J. Infect. Dis..

[51] Lewis R.E., Cahyame-Zuniga L., Leventakos K., Chamilos G., Ben-Ami R., Tamboli P., Tarrand J., Bodey G.P., Luna M., Kontoyiannis D.P. (2013). Epidemiology and Sites of Involvement of Invasive Fungal Infections in Patients with Haematological Malignancies: A 20-Year Autopsy Study. Mycoses.

[52] Nesher L., Rolston K.V.I. (2013). Neutropenic Enterocolitis, a Growing Concern in the Era of Widespread Use of Aggressive Chemotherapy. Clin. Infect. Dis..

[53] Cornely O.A., Alastruey-Izquierdo A., Arenz D., Chen S.C.A., Dannaoui E., Hochhegger B., Hoenigl M., Jensen H.E., Lagrou K., Lewis R.E. (2019). Global Guideline for the Diagnosis and Management of Mucormycosis: An Initiative of the European Confederation of Medical Mycology in Cooperation with the Mycoses Study Group Education and Research Consortium. Lancet Infect. Dis..

[54] Ismail M.H., Hodkinson H.J., Setzen G., Sofianos C., Hale M.J. (1990). Gastric Mucormycosis. Trop. Gastroenterol..

[55] Cole G.T., Halawa A.A., Anaissie E.J. (1996). The Role of the Gastrointestinal Tract in Hematogenous Candidiasis: From the Laboratory to the Bedside. Clin. Infect. Dis..

[56] Masood A., Sallah S. (2005). Chronic Disseminated Candidiasis in Patients with Acute Leukemia: Emphasis on Diagnostic Definition and Treatment. Leuk. Res..

[57] Safiia J., Díaz M.A., Alshaker H., Atallah C.J., Sakr P., Moshovitis D.G., Nawlo A., Franceschi A.E., Liakos A., Koo S. (2024). Recent Advances in Diagnostic Approaches for Mucormycosis. J. Fungi.

[58] Lamoth F., Kontoyiannis D.P. (2024). PCR Diagnostic Platforms for Non-*Aspergillus* Mold Infections: Ready for Routine Implementation in the Clinic?. Expert. Rev. Mol. Diagn..

[59] Bellanger A.-P., Gbaguidi-Haore H., Berceanu A., Gouzien L., El Machhour C., Bichard D., Lanternier F., Scherer E., Millon L., Chouaki T. (2024). Use of the Mucorales QPCR on Blood to Screen High-Risk Hematology Patients Is Associated with Better Survival. Med. Mycol..

[60] Legrand M., Gits-Muselli M., Boutin L., Garcia-Hermoso D., Maurel V., Soussi S., Benyamina M., Ferry A., Chaussard M., Hamane S. (2016). Detection of Circulating Mucorales DNA in Critically Ill Burn Patients: Preliminary Report of a Screening Strategy for Early Diagnosis and Treatment. Clin. Infect. Dis..

[61] Millon L., Caillot D., Berceanu A., Bretagne S., Lanternier F., Morio F., Letscher-Bru V., Dalle F., Denis B., Alanio A. (2022). Evaluation of Serum Mucorales Polymerase Chain Reaction (PCR) for the Diagnosis of Mucormycoses: The MODIMUCOR Prospective Trial. Clin. Infect. Dis..

[62] Moreno A., Mah J., Budvytiene I., Ho D.Y., Schwenk H.T., Banaei N. (2024). Dynamics and Prognostic Value of Plasma Cell-Free DNA PCR in Patients with Invasive Aspergillosis and Mucormycosis. J. Clin. Microbiol..

[63] Lieu A., Zimmet A.N., Pozdol J., Kushner L.E., Ho D., Banaei N. (2025). Concordance of Non-Invasive Plasma Cell-Free DNA with Invasive Diagnostics for Diagnosis of Invasive Fungal Disease. Clin. Infect. Dis..

[64] Huygens S., Schauwvlieghe A., Wlazlo N., Moors I., Boelens J., Reynders M., Chong G.-L., Klaassen C.H.W., Rijnders B.J.A. (2024). Diagnostic Value of Microbial Cell-Free DNA Sequencing for Suspected Invasive Fungal Infections: A Retrospective Multicenter Cohort Study. Open Forum Infect. Dis..

[65] Babady N.E., Chiu C.Y., Craney A., Gaston D.C., Hicklen R.S., Hogan C.A., John T.M., Stewart A.G. (2024). Diagnosis and Management of Invasive Fungal Diseases by Next-Generation Sequencing: Are We There Yet?. Expert. Rev. Mol. Diagn..

[66] Kontoyiannis D.P., Lewis R.E. (2011). How I Treat Mucormycosis. Blood.

[67] Sigera L.S.M., Denning D.W. (2024). A Systematic Review of the Therapeutic Outcome of Mucormycosis. Open Forum Infect. Dis..

